# Eliciting social stressors, supports, and determinants of health through the DSM-5 cultural formulation interview

**DOI:** 10.3389/fpsyt.2023.1148170

**Published:** 2023-03-28

**Authors:** Neil Krishan Aggarwal, Daniel Chen, Roberto Lewis-Fernández

**Affiliations:** ^1^Department of Psychiatry, New York State Psychiatric Institute, Columbia University, New York, NY, United States; ^2^Department of Psychiatry, Flushing Hospital Medical Center, Flushing, NY, United States

**Keywords:** cultural formulation, cultural formulation interview, social supports, social stressors, social determinants of health

## Abstract

**Objective:**

As recognition of the importance of social determinants of mental health has increased, the limitations of clinical competence-enhancing interventions that do not emphasize this approach have emerged. The Cultural Formulation Interview (CFI) is a cultural competence intervention that emerges from a confluence of social medicine and medical anthropology traditions. Limited research has examined how patients respond to CFI questions on social-structural aspects of illness and care to assess whether the CFI adequately elicits information on social determinants of mental health.

**Methods:**

Patients’ responses during a first intake appointment to three CFI questions on social stressors, supports, or barriers to care from 27 patient-clinician dyads are analyzed through qualitative content analysis. The data come from a hyper-diverse clinical setting in Queens, New York, where no ethnoracial group has a majority and all patient-clinician dyads reflect cross-cultural interactions.

**Results:**

At least one social determinant was coded in 89 of all cases, and nearly 44% included themes related to multiple determinants of health. The most-commonly coded theme was *social relationships* (*n* = 21), followed by *financial instability* (*n* = 7), *stigma* (*n* = 5), *housing instability* (*n* = 2), and *poor access to healthcare, involvement in the criminal justice system, employment instability*, *area-level poverty,* and *immigration policies* (*n* = 1 each).

**Conclusion:**

Our work shows that social determinants of mental health can be elicited through the CFI. Future work should examine how this information is included in clinicians’ formulations and whether the cultural formulation approach would benefit from additional revision to facilitate assessment of socio-structural factors.

## 1. Introduction

Two intellectual strands seeking to situate psychiatric symptoms within sociocultural contexts have recently converged in research on the psychiatric cultural formulation. One line comes from interpretive medical anthropologists who have questioned whether higher rates of functional impairments reported in US-born minoritized ethnoracial individuals reflect truly worse levels of functioning or biases in clinician judgments (1). Patient-clinician communication occurs across differences in cultural identities, socioeconomic class, power relations, values, and illness explanations, and symptom narratives can vary whether patients are speaking to family members or friends (1). To place symptoms within the life contexts of patients, DSM-IV’s Outline for Cultural Formulation (OCF) drew upon studies from psychiatric anthropology and social psychiatry (2) in encouraging clinicians to organize information on social stressors, social supports, and level of functioning/disability under a domain known as “Cultural Factors Related to Psychosocial Environment and Levels of Functioning” (3). In revising DSM-IV-TR to DSM-5, the DSM-5 Cross-Cultural Issues Subgroup reviewed all studies on cross-cultural assessment and the OCF since DSM-IV’s publication in 1994, finding that migration, socioeconomic status, housing, employment, residency status, language use, discrimination, acculturation, nostalgia, and biculturality were additional social stressors and supports that clinicians should consider (4). The Cultural Formulation Interview (CFI), published in 2013 for DSM-5, now includes three questions for clinicians to ask patients about social stressors, supports, and barriers to help seeking/access to care (5). Psychiatric educators have championed the OCF (6, 7) and the CFI (8, 9) as tools that can improve clinician cultural competence. Although there is no single definition for cultural competence, one widely used definition is “a set of congruent behaviors, attitudes, and policies that come together in a system or agency or among professionals and enables that system, agency, or those professionals to work effectively in cross cultural situations” (10, p. 13). Hence, social stressors, supports, and barriers to help seeking have become critical components of cultural competence initiatives in mental health.

A revitalization of social medicine scholarship has recently focused on how social circumstances affect individual health and produce disparities in health outcomes for different groups of people. Internationally, the World Health Organization (WHO) undertook a campaign to synthesize information on social and economic determinants of health for policy makers (11). The US Centers for Disease Control and Prevention (CDC) defines social determinants of health as “life-enhancing resources, such as food supply, housing, economic and social relationships, transportation, education, and health care, whose distribution across populations effectively determines length and quality of life” (12, p. 6). In the US, two influential reports connected public health knowledge to disparities in mental health outcomes for minoritized ethnoracial individuals. In 2001, the US Surgeon General released *Mental Health: Culture, Race, and Ethnicity* to document worse psychiatric outcomes for minoritized ethnoracial groups compared to non-Latinx White people that could be traced to differences in the housing, transportation, employment, criminal justice, and educational sectors (13). In 2002, the National Academy of Sciences released *Unequal Treatment: Confronting Racial and Ethnic Disparities in Health Care,* showing that minoritized ethnoracial groups experience worse health outcomes than White people due to differences in socioeconomic status, language barriers, geographical segregation, and financial stability (14). The WHO now lists dozens of resources on its website to encourage national government policy makers to address social determinants of health outside of clinical settings.^1^ Similarly, the CDC has identified social determinants as a “priority area” on its website with dozens of resources for organizations outside of the health sector to assist in identifying, measuring, and alleviating inequities.^2^ Neither organization offers specific guidance to mental health clinicians on how to assess and address the impact of social determinants on their individual patients.

To address these social determinants, some psychiatrists have recommended that clinicians develop “structural competence,” defined as “the trained ability to discern how a host of issues defined clinically as symptoms, attitudes, or diseases… also represent the downstream implications of a number of upstream decisions about such matters as health care and food delivery systems, zoning laws, urban and rural infrastructures, medicalization, or even about the very definitions of illness and health” (15, p. 128). Within structural competence, structure is conceived as “the buildings, energy networks, water, sewage, food and waste distribution systems, highways, airline, train and road complexes, and electronic communications systems that are concomitantly local and global” (15, p. 128). Structural competence’s advocates have not provided definitions for the words “structure” or “culture” (15, 16), but they argue that both concepts are separate, as this passage suggests:

“Social scientists and humanities scholars add important conceptualizations of structure as a system that produces and reproduces the social world, and that is thus deeply linked to culture because it provides the system of values affixed to bodies and diseases. And political and public-health activists use structures of oppression, such as racism or debt, to address seemingly biological conditions of morbidity and mortality. Calling on these and other literatures, structural competency seeks to promote skills, not so much for replacing awareness of “culture” in medical settings, but for recognizing how “culture” and “structure” are mutually complicated in producing stigma and inequality” (15, p. 128).

A debate has emerged about whether social medicine approaches (“structural competence”) should be emphasized over interpretive medical anthropology approaches (glossed as “cultural competence”). Advocates for this position critique cultural competence interventions for usually training clinicians about the beliefs and behaviors of individual patients facing inequities predominantly in terms of ethnic identity, resulting in interventions that ignore the sources of such inequities (15–17). They argue that clinicians must rearticulate cultural formulations in structural terms to recognize how social, economic, and political conditions produce health inequalities (16). Others argue that the two traditions offer complementary information about a patient’s identity, life experiences, and suffering that can be combined to guide clinical interventions (18). This debate raises broader questions not just in psychiatry but within all of health care about how clinicians can deliver patient-centered care that responds to each individual’s unique needs while situating all individuals within social determinants of health.

The OCF and CFI emerge from a confluence of approaches from social medicine and interpretive medical anthropology. The question remains whether the OCF adequately includes a social medicine perspective and whether the CFI elicits socio-structural information sufficiently *via* open-ended questions about sufferers’ views of their own experience. Structural factors may be at a level of abstraction that many patients are uncomfortable discussing, especially because they know this content is typically not what clinicians consider germane to their assessment. Given the value of both traditions, some have suggested expanding the OCF to focus more on structural factors, resulting in a revised sociocultural formulation (18). To our knowledge, patient-elicited narratives obtained with the OCF or the CFI have never been examined to determine if and to what extent either tool elicits social determinants of mental health. It is possible that the CFI elicits certain social determinants of health in its current form – for example, by asking about barriers to care – but that it would need to be revised to address others. The aims of this paper are to (1) explore how patients responded to CFI questions on social stressors, supports, barriers to help seeking, and access to care, and (2) analyze patient responses according to an emerging framework on the social determinants of health. Answers to these questions can clarify the extent to which the CFI adequately captures domains relevant to social determinants of health.

## 2. Methods

### 2.1. Setting

The data presented here come from a larger clinical and ethnographic study on patient-clinician communication at an outpatient mental health clinic that receives referrals from 2 tertiary-care hospitals through a community network that we call Crown Health Systems (CHS) in Queens, New York. This clinic operated from 9am to 9pm on Mondays through Fridays and 9am to 12pm on Saturdays. According to service administrators, evening and weekend hours increase the likelihood that patients will attend appointments that do not conflict with employment schedules. On staff are 30 administrators, psychiatrists, psychologists, nurses, and social workers. The clinic’s services include individual psychotherapy, group psychotherapy, couples and family counseling, and medication management for people of all ages. Social workers complete a social history assessment and provide case management. Although social determinants of health are not systematically assessed through a single questionnaire, social workers conduct assessments and refer patients for transportation vouchers, subsidized housing, food stamps, and vocational training. The clinic refers to an emergency room when patients exhibit substance intoxication, suicidal thoughts and behaviors, or homicidal thoughts and behaviors, which may lead to inpatient hospitalization at a different section of the medical campus. The outpatient clinic receives referrals from providers in family medicine, internal medicine, obstetrics/gynecology, and surgery.

We chose this site to examine how clinicians could use the CFI with patients because of its sociodemographic diversity. According to the latest 2020 US Census, Queens is the most ethno-racially diverse American urban center: ~28% identify as Latinx, 27% non-Latinx Asian, 22% non-Latinx White, 16% non-Latinx Black, and the remainder identify as belonging to other ethnoracial backgrounds (19). In 2014, when the study was initiated, the clinic had about 1,500 outpatients, and ~ 30% identified as non-Latinx White, 30% Latinx, 32% non-Latinx Asian, and 8% non-Latinx Black. At CHS, interpreters are available on site for 15 languages, and over-the-phone interpretation is available 24 h/day in over 200 languages. Each floor has information about interpreters, patient forms in preferred languages, and signs in different languages on patient rights. Patients and clinicians use Spanish, Mandarin, Cantonese, Korean, or Hindi-Urdu for conversations that do not take place as part of their formal appointments, but usually switch to English for their clinical encounters. CHS is located in an area that social scientists have characterized as “hyperdiverse,” defined as “social settings in which complex interactions among multiple forms of difference and diversity – for instance, race, ethnicity, culture, gender, sexual orientation, and socioeconomic class – intersect in ways that significantly influence efforts to cultivate clinical rapport and trust” (20, p. 20).

### 2.2. Study design

These data come from a five-year study of patient-clinician interactions. We spent over 500 h at CHS with the first author leading clinical and ethnographic data collection. The first author sought to understand everyday life at the clinic for 4 months by sitting in waiting rooms, clinical meetings, IRB meetings, and didactic lessons as clinicians taught trainees in psychiatry, psychology, and social work. Field notes were shared with the research team.

Ethnographic observations revealed the basic operations of the clinic: all patients attend a 60-min intake with a social worker or a psychologist (Session 1), a 45-min appointment with a psychiatrist (Session 2), and a 60-min first psychotherapy session with a social worker or a psychologist (Session 3) within 1 month. Clinicians agreed to implement the CFI in Session 1 after CHS’s administrators gave assurances that a CFI training session would fulfill the institution’s annual education requirements for cultural competence and all sessions would be billable.

### 2.3. Participants

Patients were included from any ethnoracial background; if they were aged 18–80; spoke English even if they had a different primary language to match their clinician’s language fluency; and presented to establish initial care. Patients were excluded if they had acute suicidality or homicidality, dementia, severe intellectual disability, or psychosis which could interfere with responding to questions. All patients received $30 each session for their time.

Clinicians who could practice with an independent license were eligible for inclusion. The only exclusion criterion was if clinicians did not agree to attend training on the CFI, described in the next section. No clinician who agreed to participate in the trial was excluded for refusing to attend the training.

### 2.4. Study design

We conducted a clinical trial of clinicians using the CFI with new outpatients. The clinic’s intake coordinator presented the study to all patients upon scheduling their first session. We followed the NIMH’s stage model to convert social science theories that are based on descriptive research into behavioral interventions by conducting a pilot study that enrolled at least 12 patients where the trial’s focus was mainly to standardize training procedures with a limited number of clinicians to ensure the intervention’s replicability (21). Our trial trained clinicians through effective practices in adult learning that replicated the training procedures from the DSM-5 field trial for the CFI, such as reading the entire CFI, watching a video on its use, practicing questions through case simulations, asking the first author questions about unclear usage, and providing feedback after the first case (22). All clinicians received CFI training before patients were enrolled. After completing fidelity ratings through the CFI-Fidelity Instrument (23), we found that all clinicians asked all patients each CFI question. No additional training sessions were needed.

All participants provided written informed consent to participate in the study, record all information, and reproduce quotes. Study approval was received from all participating institutions.

### 2.5. Data analysis

All interviews were transcribed with the first author listening to recordings and checking the transcription again by listening to recordings a second time. We created a coding scheme based on the US National Academies of Science, Engineering, and Medicine’s 2020 framework on the different types of social determinants of mental health (24, henceforth abbreviated as “US National Academies”). This coding scheme was then applied to three questions from the CFI on social stressors, supports, barriers to help seeking/access to care through content analysis:

CFI Question 6: Are there any kinds of support that make your [PROBLEM] better, such as support from family, friends, or others?

CFI Question 7: Are there any kinds of stresses that make your [PROBLEM] worse, such as difficulties with money, or family problems?

CFI Question 13: Has anything prevented you from getting the help you need? PROBE AS NEEDED: For example, money, work or family commitments, stigma or discrimination, or lack of services that understand your language or background?

We chose only to analyze direct responses to these questions, consistent with the CFI’s development as an approach to clinical interviewing that centers patient perspectives over professional biomedical knowledge (2, 3, 5). While it is possible that patients discussed social determinants at other points in the interview, these three questions pulled specifically for patients’ own assessment of stressors, supports, and barriers, probing social characteristics (e.g., difficulties with money, discrimination, lack of services). By focusing on these sections of the CFI we avoid drawing inferences about patients’ social worlds that patients themselves did not volunteer directly.

The first author created a codebook with definitions for codes from the US National Academies framework on social determinants of health (24). He coded each sentence under the 3 CFI questions to ensure that all text would be coded. He entered transcripts into NVivo and followed analytical triangulation to check codes against the coding scheme. The first author created new codes when framework-based codes did not capture patient responses. To ensure rigor and validity of our analyses, the first and third authors held team debriefing sessions, and the first author drafted analytical memos. The third author checked the first author’s coding so that inter-rater reliability exceeded 90%, with discrepancies resolved through consensus with co-authors. The first and third co-authors are both cultural psychiatrists with additional training in clinically applied medical anthropology who each have over a decade of experience with qualitative and mixed-methods data analysis.

## 3. Results

### 3.1. Sample characteristics

Twenty-seven patients enrolled. Ten identified as male and 17 as female. Their ages ranged from 18 to 67 years (mean = 32.8, SD = 14). Three identified as non-Latinx White, 12 as Latinx, 9 as non-Latinx Black, and 3 as non-Latinx Asian. Patient primary languages were English (*n* = 21), Spanish (*n* = 5), and Polish (*n* = 1). Their primary sources of income were employment (*n* = 12), family assistance (*n* = 5), disability (*n* = 3), public assistance (*n* = 3), and pension (*n* = 1); 3 patients chose not to respond about their sources of income. One patient earned $120,000 in annual income and the rest earned $60,000 or less annually. Their primary intake diagnoses by DSM-5 diagnostic class were depressive (*n* = 11), anxiety (*n* = 9), bipolar (*n* = 3), adjustment (*n* = 2), psychotic (*n* = 1), and somatic-symptom (*n* = 1) disorders.

Two clinicians enrolled in the study. Both were social workers. One identified as male, the other as female, and both as non-Latinx White. The male therapist was a native citizen of the United States and the female therapist was a naturalized American citizen who was born in Central Asia. Neither had any experience with the CFI prior to the study.

### 3.2. Social stressors, supports, and determinants of health themes

All 27 patients provided answers to the 3 CFI questions, indicating that they understood the questions. Three patients (11%) indicated that they did not experience social stressors, social supports, or barriers to help seeking/access to care. The remaining 24 patients (89%) provided answers that were coded by theme. Table 1 presents themes on the social determinants of health as proposed by the US National Academies and captured in our coding.

**Table 1:** Themes on social supports, stressors, and determinants of health in response to three cultural formulation interview questions analyzed from qualitative interviews (*N* = 27 patient-clinician dyads).

Theme	National academies framework	Coded in our analyses
Homelessness/housing instability	Yes	Yes
Food insecurity	Yes	No
Transportation insecurity	Yes	No
Poor access to health care	Yes	Yes
Adverse early life experiences	Yes	No
Discrimination	Yes	No
Exposure to violence/conflict	Yes	No
Criminal justice involvement	Yes	Yes
Adverse infrastructure	Yes	No
Neighborhood disorder	Yes	No
Pollution exposure	Yes	No
Climate change	Yes	No
Low education	Yes	Yes
Employment instability	Yes	Yes
Financial instability	Yes	Yes
Area-level poverty	Yes	Yes
Social relationships	No	Yes
Stigma	No	Yes
Immigration policies	No	Yes

The most common themes from the National Academies Framework were *financial instability* (*n* = 7) and *housing instability* (*n* = 2). Certain themes from this Framework appeared in the narrative of only a single patient, such as *poor access to healthcare, involvement in the criminal justice system, employment instability*, and *area-level poverty*.

The US National Academies included multiple themes for which we could not find instances to code, such as *food insecurity, transportation insecurity, adverse early-life experiences, exposure to violence/conflict, adverse features of infrastructure, neighborhood disorder, pollution exposure,* and *climate change*. It is possible that these social determinants existed for patients who might have been less likely to report them during the CFI of an initial session, perhaps until sufficient rapport was built with clinicians. It is also possible that these social determinants existed, but patients did not see their direct relevance to their illnesses or answer them under the questions we analyzed. For instance, patients might have been aware that symptoms of anxiety, mood, or traumatic stress disorders could develop from *exposure to violence/conflict*, but may not have made an explicit link to other social determinants such as *adverse features of infrastructure, neighborhood disorder, pollution exposure,* and *climate change*.

Our coding produced three social determinants of health that were not in the US National Academies’ 2020 framework. The most-commonly coded theme was *social relationships* (*n* = 21), followed by *stigma* (*n* = 5), and *immigration policies* (*n* = 1). We created these codes after reviewing older frameworks on the social determinants of health that included these themes (12–14). For example, a 2018 systematic review of literature reviews on social determinants of health identified 289 articles, resulting in a conceptual framework of five domains: demographic (e.g., community diversity), economic (e.g., economic inequality), neighborhood (e.g., safety/security), environmental events (e.g., war/conflict), and social and cultural (e.g., social support) (25). Other examples of this fifth domain included individual social capital, social participation, and social stability; our theme of social relationships falls squarely in this domain. In an earlier publication, the US National Academies acknowledged multiple frameworks on the social determinants of health, observing no single scholarly consensus (26).

Twelve patients named multiple social determinants of health, with *social relationships* appearing in each combination.

Due to spatial constraints, we present qualitative data with representative patient quotations for all themes that appeared in more than one patient response.

#### 3.2.1. Social relationships

Twenty-one patients named social relationships with family members, friends, and close associates in responses that were coded as a social determinant of their mental health. After exploring how these codes were similar to and different from each other through thematic analysis, we discovered 4 subthemes: (1) patients described some relationships that were positive and others that were negative (*n* = 9), (2) patients only described positive relationships in their lives (*n* = 7), (3) patients only described negative relationships in their lives (*n* = 3), and (4) patients described the same relationships as positive and negative (*n* = 2). Each example shows how relationships determine quality of life. All the names below are pseudonyms to protect patient confidentiality.

##### 3.2.1.1. Some relationships as positive and others as negative

Mrs. Brown’s response typified how some relationships could be viewed as positive and others negative. She moved to the United States from the Caribbean as an adolescent. Now in her thirties, she established care for depression that began during her second pregnancy. She described her problem to friends as, “Sometimes I feel real down. Then there are times I feel suicidal. I’ve expressed that to my mom, because she’s the only parent that I have that is alive currently. I was very close to my dad. He passed away when I was 15 years old. I never really dealt with that, and it still affects me to this day.” She and her clinician discussed social supports and stressors:

Clinician: Are there any kinds of support that make your feeling down better? Such as support from family, friends, or others?

Mrs. Brown: When people come around, I do not feel down. I feel happy when everybody’s around me. Or I’ll speak to my mom. I’ll see her on Facetime - then I’ll be happy. But then there’s days where I just, like I think about it, that, “Oh my god, I’m here by myself. And I thought I had family that cared about me, which they do not, because they treated me real bad when I came here.”

Clinician: Meaning your paternal aunt?

Mrs. Brown: Yeah.

Clinician: Your father’s sister?

Ms. Brown: Right. So then when I see other people with their family, you know, it kind of gets me down.

Clinician: Are there any kinds of stresses that make your feeling down worse? Such as difficulties with money or family problems?

Mrs. Brown: Well, mainly with my daughter because she has cerebral palsy. And whenever we go out, I see it in her face. She wants to go play with other kids and she cannot. It really affects me a lot.

Clinician: She’s what, 8 or 9?

Patient: She’s 8.

Mrs. Brown’s narrative showed how some of her relationships were positive and others were negative. Her mother provided her with support. However, she did not forgive her paternal aunt for complicating her migration to the United States. Her father’s death in her home country while she was in the United States marked the start of her depression. Seeing her child struggle socially with peers worsen the depression.

##### 3.2.1.2. All relationships as positive

In contrast, Ms. Smith’s answers exemplified how some patients only maintained positive relationships in their lives. An eighteen-year-old born and raised in the United States, she described the reason for establishing care as “Court is coming, and I’ve got to come here to get my daughters back.” She described her problem as, “They are trying to say I’m not fit to have my kids when I’m obviously fit. Like, nothing is wrong with me. The courts are the problem. ACS [Administration for Children’s Services] is the problem.” Her daughters were both under the age of 18 months, and she explained how her children were removed from her legal custody in vague terms, “I took one of my daughters to my ex’s house, and we got into an altercation. And then, one thing led to another and now I’m here.” The clinician asked her about social stressors and supports:

Clinician: Are there any kinds of support that make your court issue better? Such as support from your family, friends, or others?

Ms. Brown: Yes. I got support from my family and my friends. I already got friends, but all the people around me.

Clinician: And are there any kind of stresses that make your court issue worse such as difficulty with money or family problems?

Ms. Brown: I would say traveling. Traveling be the worst. But other than that everything is going good for me right now. So it’s not really bad.

For Ms. Brown, her ex-boyfriend was a negative relationship from her past. She refused to interact with him once their legal dispute began. After family court assumed custody of her children, he ceased to be relevant in her life. She emphasized that she only spent time with people who cared about her.

##### 3.2.1.3. All relationships as negative

Ms. Vásquez’s answers typify how some patients only had negative relationships. She was a woman in her forties who established care to continue receiving antidepressant medication which her primary care physician had prescribed. She described her problem as “I just keep to myself. I like to lay down or I just keep quiet. I do not want anybody talking to me.” When her clinician asked what troubled her most about the depression, she replied, “It’s interfering with a lot in my life. I do get some things done, but not all of it like I should. I do not want to do it half of the time. So it’s not good.” Her clinician asked her about the cause of depression, and Ms. Vásquez responded, “I think it’s several things; not just one. Number one is being a single mother.” Her clinician then asked her about social supports and stressors:

Clinician: And are there any kinds of support that make your issues, your depression better/ such as money, family problems, anything?

Ms. Vásquez: Well, what would make it worse is family problems.

Clinician: How are you doing raising your son? Is that stressful? Or is that okay? Do you feel like you are handling being a single parent?

Ms. Vásquez: I’m with him, but I do not show it. I feel inadequate.

Clinician: Your depression or your lack of motivation?

Ms. Vásquez: Well, part of that is because I feel that I’m not a good mom because I cannot support him the way I want to. That’s a huge problem.

Clinician: So, you want to be able to provide him with more?

Ms. Vásquez: Yes, with more, and the most painful part of it all is he throws it in my face. He’s very mean about it.

Ms. Vásquez did not name other social relationships during her intake appointment. Her narrative shows how negative social relationships can trigger depressive symptoms which are then maintained as long as those relationships are not improved.

##### 3.2.1.4. The same relationships as positive and negative

Only two people provided responses that could be coded in this manner. Of them, Ms. Williams was a Black woman in her fifties. When asked why she wanted to establish care, she said, “I came here today because I would like to continue therapy. I graduated a treatment center for alcohol. I’ve been in the process of getting a new therapist and getting a new doctor. I have mental health issues and I’m on medication.” She had gotten engaged earlier that year. When her clinician asked her about social stressors and supports, she named her fiancé in both answers:

Clinician: Are there any kinds of support that make bipolar better such as support from family, friends or others?

Ms. Williams: Yeah, support from my significant other. He’s a big support.

Clinician: Are there any kinds of stresses that make your bipolar worse such as difficulties with money or family problems?

Ms. Williams: Sometimes I have disputes with my fiancé and since we live in two separate places, I’ll remove myself from the situations that bring me stress from like anybody. I put my health first. I put me first.

Ms. William experienced three major hospitalizations over 10 years of treatment. She described few close relationships at this point in her life. Nonetheless, her biggest source of support also acted as her biggest stressor.

#### 3.2.2. Financial instability

Mr. Sánchez’s answers typified the responses of all seven patients who identified financial instability as a problem. A Latinx man in his late forties, he had sought treatment inconsistently for a psychotic disorder over the preceding 3 years. When his clinician asked him why he wanted to establish care, Mr. Sánchez said, “I need to let a couple things out of my chest. I need to speak about things. Because if I hold it back, it just – I end up going to the same place, end up doing the same thing, which always ends up in nothing. It’s just – for some reason, I cannot function. I cannot do nothing good. I think about a lot of things that I want to do. And the things - most of the things that I want to do is just sort of like – it’s a dream. Like sometimes I picture myself with a lot of money and doing other - acting like another person. Just – it’s just a way of feeling good about myself.” They had this exchange about social stressors and supports:

Clinician: Are there any kinds of support that make your problem better, such as support from family, friends, or others?

Mr. Sánchez: No, I do not get that a lot.

Clinician: Are there any kinds of stresses that make your problem worse? Such as difficulties with money or family problems?

Mr. Sánchez: Everybody in my family – I would say they are doing better than I am. This week, we went to celebrate my sister’s birthday. Everybody put up money except for me. I had $3 with me. I took it out, but I guess they did not want to take it. They did not want to take nothing. They must have thought that I do not got not money. I was making a joke, but deep inside, I wasn’t happy about it. I just wanted to go outside and say, “I’ll be back in a minute.” But I was going to walk out. I was going to leave because I do not belong there.

His clinician asked about barriers to care:

Clinician: Has anything prevented you from getting the help you need? For example, money, work, or family commitments, stigma or discrimination, or lack of services that understand your language or background?

Mr. Sánchez: Money is a problem. Money is important, but I do not do much with money. I do not get a lot of things with money. I do not know what’s the cure for me. I do not even know why I cannot be like everybody else. I cannot fit in.

In Mr. Sánchez’s view, financial problems caused his difficulties. He described emotional distress at not having enough money in a social setting with his relatives. He also named financial problems as barrier to receiving health care. His narrative indicates how money was a resource that determined his quality of life in an ongoing manner.

#### 3.2.3. Stigma

Stigma is not in the US National Academies framework but is included in other frameworks on the social determinants of health (15–17, 26). In our dataset, stigma appeared as a theme in 5 narratives. Therefore, we used a definition for stigma as experiencing a health condition that is viewed by oneself and/or others as socially deviant (27).

Ms. García’s responses were similar to the other four patients. When her clinician asked why she wanted to establish care, Ms. García said, “My husband passed away, I’m sorry [starting to cry], it’s been over 3 years and I still—I cannot do it. I cannot. And I feel hopeless. He was my comfort, my rock. I’ve always dealt with anxiety and he would—without saying a word, he would just take my hand or hug me and calm me down or walk me around the park. You know. He’s not there anymore and my anxiety is getting worse.” They discussed her barriers to care:

Clinician: Has anything prevented you from getting the help you need? For example, money, family commitments, stigma, or discrimination or lack of services that understand your language or background?

Ms. García: I guess it would be in the back of your head that stigma of, you know, you can work things out yourself. That you do not need that kind of help. People talk about mental illness, and I kind of do not need that.

Like other patients who reported this theme, Ms. García viewed her symptoms of anxiety as a mental illness for which she did not need help. Stigma delayed her entry into mental health care by over 3 years. Stigma acted as a social determinant of health in postponing treatment, an essential resource that determines quality of life.

#### 3.2.4. Housing instability

Two patients identified housing instability as a social determinant of their mental health. In both cases, housing instability appeared in the context of difficulties in social relationships. For instance, Ms. Khan was a single woman in her early twenties who was born in Pakistan. She attended a local community college at the time she initiated care for depression. Her parents went through a bitter separation, and she chose to live with her mother. When the clinician asked her what brought her to the clinic, she said, “After my mother, I’m the primary caregiver of my two special-needs brothers. With their growing age and my college, that stress is beginning to get the best of me. And also, unfortunately, these past few years my relationship with my father has deteriorated. So that itself also has played a role within these changes. It’s come to a point where I sometimes cannot understand what is stressing me out or bothering me at that moment. A mood swing can happen quite literally at any moment.”

Her clinician asked about how she perceived the causes of her depression, and Ms. Khan provided details about her relationships: “My two younger brothers, 16 and 17 years old. They’re both autistic and non-verbal. My deteriorating relationship with my father, unfortunately, and at this point not being able to balance well my school life and my personal life.”

The CFI’s questions on social stressors and supports illuminated how this deteriorating relationship with her father led to housing instability:

Clinician: Are there any kind of supports that make your depression, your mood swings better? Such as support form family, friends or others?

Ms. Khan: My mother.

Clinician: Your mother is a big support?

Ms. Khan: Yes.

Clinician: Are there any kind of stresses that make your depression and your mood swings worse such as difficulties with money or family problems?

Ms. Khan: Very much.

Clinician: How so?

Patient: When it comes to the money aspects, especially rent. We lived in Brooklyn before. When my father had separated, we had come to know that he had not been paying the rent regularly and that lead to an eviction court case. Luckily through that, we were able to get an approval for a voucher, a rental assistance voucher. A lot of times if there is consistently missing rent, because it’s connected to a public assistance case, the minute someone starts doing a job, they are taken off assistance and that’s slashes the amount of the voucher. Then, when you visit NYCHA [New York City Housing Authority], people do not seem to take your case seriously or do not seem much interested in it.

In Ms. Khan’s narrative, depression and mood swings emerged from challenging relationships with two generations of male family members. The accumulation of situational stressors – attending college, caring for brothers with autism spectrum disorder, and imminent eviction – disturbed her emotional balance to such an extent that mood swings manifested unpredictably. Without public assistance, her household would be unhoused. But with public assistance, there was a limit to how much she could earn through employment without losing her domicile. Hence, housing was an essential resource that determined her quality of life and mental wellbeing.

## 4. Discussion

This study has analyzed patient narratives through the CFI to determine the extent to which social determinants of mental health can be elicited. At least one social determinant was coded in close to 90% of all cases, and nearly 44% (*n* = 12) included themes related to multiple determinants of health. To our knowledge, this is the first study on the CFI that reports empirical data specifically on the social determinants of health from a clinical sample.

Our study location could explain why we coded certain social determinants of health but not others. CHS is located in an area of Queens with a predominantly immigrant and minoritized ethnoracial population. Patient demographics show that most individuals had lower household incomes. Certain themes such as *housing instability, poor access to health care, employment instability, financial instability*, *area-level poverty*, and *immigration policies* could be due to lower disposable incomes. We did not find instances of *food insecurity* or *transportation insecurity*. Perhaps patients could afford food or transportation or relied on public assistance from New York City. At the same time, our clinicians did not follow up with patients pertaining these themes, so we cannot speculate on the relationship between household socioeconomic status and specific social determinants of health. Time constraints affect all real-world implementation of the CFI and attempts to use the CFI to elicit social determinants of health may need to be addressed during training for clinicians along with ways for clinicians to bill for time spent with patients.

There are certain themes which we expected to code, but for which we did not find instances in the three CFI questions we examined. Some of these themes may have been elicited by other CFI questions. For example, in a different part of the interview, Mrs. Brown alluded to the death of her father when she was 15 years old, which can be considered an instance of *adverse early-life experiences*. In describing her reasons for coming to care, Ms. Smith attributed her visit to distressing interactions with the court system and ACS. We lack information to determine what aspects of these interactions were particularly distressing; for example, was she hinting at experiences of *discrimination* or simply describing general difficulties making her case to government authorities? Some patients may test clinicians’ reactions with brief comments meant to elicit follow-up questions to determine whether the clinical space is “safe” to present certain points of view. Ms. Smith’s comments were not explored by the clinician and the interview moved on to other topics. A limitation of our targeted approach is that patients may interweave descriptions of social determinants into life narratives and pursue other responses when asked directly about social stressors or supports. Future research can disentangle these possibilities. However, additional reports of social determinants throughout the CFI further would support our finding that the CFI elicits this information. Interestingly, we did not encounter narratives of *discrimination* in the items we coded. Perhaps patients did not experience this on a regular basis given the neighborhood’s diversity. It is also possible that patients experienced discrimination but did not name this in an initial session. Moreover, we did not encounter narratives of *exposure to violence/conflict*, and no patient received a DSM-5 diagnosis of a stressor or trauma-related disorder. It is possible that patients with such disorders chose not to be included in our study.

The results of our study have broader implications for scholarship in the social determinants of health. There is currently no method that clarifies which social determinants should receive priority in improving care for mental disorders in a particular location and for which populations interventions are most effective despite growing calls to address the social determinants of mental health (28). It is likely that no single typology will address all circumstances for all people given that economic, political and social structures differentially distribute resources across populations (11–15). With this assumption, social determinants of mental health could be expected to differ across clinical settings. Still, repeated assessments of social determinants with the same population over time may reveal which interventions are most likely to improve mental disorders. Our work indicates that clinicians can use the CFI to offer patient-centered care that situates individuals within the matrix of social determinants that they deem most pertinent to their current illness episode.

This study has several limitations. First, our study has limited sample size given its exploratory nature. Nonetheless, methodologists in qualitative research suggest that data saturation can be achieved with 12 interviews, and basic meta-themes are revealed with 6 interviews (29). Our dataset of 27 interviews suggests that we achieved data saturation on social determinants of mental health with this sample; later-coded interviews elicited no additional codes. Second, the question structure of the CFI could have influenced how patients responded. For instance, examples and probes included in CFI questions on social supports, stressors, and barriers to care all include certain themes but not others, and this could explain why patients named *social relationships* and *financial problems* most frequently. At the same time, patients named other themes, so examples in CFI questions appeared to open a conversational space rather than restrict options. More research is needed as to whether additional examples should be added if revisions to the CFI are contemplated. Third, clinicians did not ask follow-up questions to probe for additional social determinants of health beyond those reported. This exploratory study emphasized interviewer fidelity to the CFI and sought to limit its duration to fit standard CHS procedures for an initial assessment. The CFI fidelity instrument used penalized clinicians for drifting the medical interview away from the core CFI’s questions (23). Future assessments of fidelity could benefit from a more expansive understanding that balances clinician competence in asking questions with drift if probe questions are asked.

Despite these limitations, our work shows that social determinants of health can be elicited through the CFI. This corresponds to the impact of both social medicine and medical anthropology in the development of the instrument. Published in 2022, DSM-5-TR encourages clinicians to identify key stressors, challenges, and supports in an individual’s environment as social determinants of mental health (30), and our study demonstrates that the CFI can be used for this purpose. Ultimately, debate about whether structural formulations should replace cultural formulations or whether both can be synthesized raises questions about how clinicians construct formulations in the first place. No current work systematically evaluates how clinicians construct formulations, a task that we propose for future investigators.

## Data availability statement

The original contributions presented in the study are included in the article/supplementary material, further inquiries can be directed to the corresponding author.

## Ethics statement

The studies involving human participants were reviewed and approved by the New York State Psychiatric Institute. The patients/participants provided their written informed consent to participate in this study. Written informed consent was obtained from the individual(s) for the publication of any potentially identifiable images or data included in this article.

## Author contributions

NA generated the data and drafted the first manuscript. NA and RL-F designed the study and analyzed the data. All authors contributed to the article and approved the submitted version.

## Funding

This work is supported by a grant from the National Institute of Mental Health (#MH102334) to NA. The senior author receives institutional support from the New York State Psychiatric Institute and NA receives support from a contract with the New York State Office of Mental Health.

## Conflict of interest

The authors declare that the research was conducted in the absence of any commercial or financial relationships that could be construed as a potential conflict of interest.

## Publisher’s note

All claims expressed in this article are solely those of the authors and do not necessarily represent those of their affiliated organizations, or those of the publisher, the editors and the reviewers. Any product that may be evaluated in this article, or claim that may be made by its manufacturer, is not guaranteed or endorsed by the publisher.

## Footnotes

^1^ https://www.who.int/health-topics/universal-health-coverage/social-determinants-of-health#tab=tab_3

^2^ https://health.gov/healthypeople/priority-areas/social-determinants-health
